# Prospective assessment of probe‐based confocal laser endomicroscopy under direct cholangioscopic visualization for biliary strictures that could not be definitively diagnosed using endoscopic retrograde cholangiopancreatography (with video)

**DOI:** 10.1002/deo2.70007

**Published:** 2024-09-25

**Authors:** Yuki Tanisaka, Shomei Ryozawa, Masafumi Mizuide, Akashi Fujita, Ryuhei Jinushi, Ryuichi Watanabe, Ryo Sato, Tomoaki Tashima, Yumi Mashimo

**Affiliations:** ^1^ Department of Gastroenterology Saitama Medical University International Medical Center Saitama Japan

**Keywords:** biliary stricture, biliary tract neoplasms, cholangioscopy, confocal laser microscopy, endoscopic retrograde cholangiopancreatography

## Abstract

The definitive diagnosis of patients with indeterminate biliary strictures remains challenging. Probe‐based confocal laser endomicroscopy (pCLE) provides real‐time histological assessment of bile duct tissues. Since no previous studies have evaluated the efficacy of pCLE under direct cholangioscopic visualization for biliary strictures that cannot be definitively diagnosed through endoscopic retrograde cholangiopancreatography using fluoroscopy, we prospectively assessed the feasibility and safety of this procedure in three cases. pCLE findings were obtained in three cases, providing accurate diagnoses. Additionally, no adverse event was reported. pCLE under direct cholangioscopic visualization for indeterminate biliary strictures might be feasible and safe, even though these strictures were not previously diagnosed using endoscopic retrograde cholangiopancreatography. Further studies with more cases are warranted to clarify the effectiveness of pCLE under direct cholangioscopic visualization.

## INTRODUCTION

Endoscopic retrograde cholangiopancreatography (ERCP) using fluoroscopy is considered the gold standard and the first step. However, its diagnostic yield has been reported as unsatisfactory.^1^ Probe‐based confocal laser endomicroscopy (pCLE; CholangioFlex; Cellvizio; Mauna Kea Technologies) has been used to differentiate benign from malignant biliary strictures.^2^ A recent meta‐analysis reported that the pooled sensitivity and specificity were 88% and 79%, respectively.^3^ Furthermore, the usefulness of pCLE under direct cholangioscopic visualization has been reported as the confocal mini‐probe can accurately be applied to the stricture area.^4^, ^5^ We aimed to prospectively evaluate the feasibility and safety of pCLE under direct cholangioscopic visualization for biliary strictures that cannot be definitively diagnosed by ERCP.

## CASE REPORT

In this prospective case series, we conducted pCLE under direct cholangioscopic visualization in three patients with biliary strictures that could not be definitively diagnosed through ERCP using fluoroscopy. We defined the period for performing this procedure from July 2023 to March 2026, which was approved by the Certified Clinical Research Review Committee (number: 232001). It has been registered in the Japan Registry of Clinical Trials (jRCT; number: jRCTs031230238). Written informed consent was obtained before the procedure. As a result, we performed three procedures in August 2023, October 2023, and November 2023.

## pCLE UNDER DIRECT CHOLANGIOSCOPIC VISUALIZATION

**FIGURE 1:**
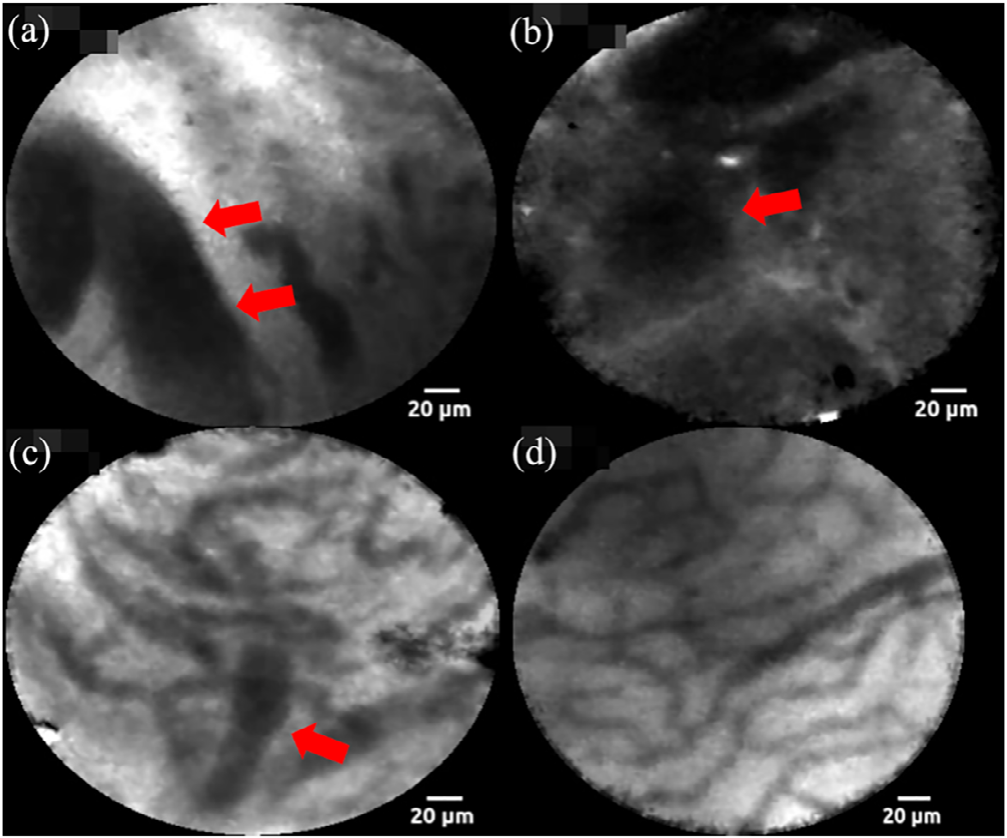
pCLE findings evaluated using the Miami and Paris classifications.^8^, ^9^ (a) Thick dark bands (>40 µm; red arrow) in the Miami classification. (b) Dark clumps (red arrow) in the Miami classification. (c) Thickened reticular structure (red arrow) in the Paris classification. (d) Reticular network of thin dark branching bands (<20 µm) in the Miami classification. pCLE, probe‐based confocal laser endomicroscopy.

All procedures were performed under conscious sedation. After selective biliary cannulation, cholangiography was performed to visualize the biliary stricture, followed by cholangioscopy using a digital single‐operator peroral cholangioscope (POCS; SPY‐DS, SpyGlass DS; Boston Scientific Corp.).^6^ Subsequently, intravenous fluorescein 0.5 mg/kg was injected,^7^ and pCLE was performed under direct cholangioscopic visualization. Thereafter, the confocal mini‐probe was inserted through the forceps channel of the POCS and gently applied to the bile duct strictures to capture confocal images. The pCLE images were interpreted prospectively using the Miami and Paris classifications (Figure 1a–d and Table S1) in real time.^8^, ^9^ After pCLE, tissue acquisition was performed using SpyBite MAX (Boston Scientific Corp.) under direct cholangioscopic visualization.

Two investigators (Akashi Fujita and Ryuhei Jinushi) who had previously been trained in pCLE image interpretation and were not involved in procedures in real‐time were asked to review each case and record the presumptive diagnosis based on the pCLE findings. They were blinded to the final diagnosis. A final diagnosis was made by pathological findings or clinical course. A final diagnosis of malignancy was made based on pathological findings. A final diagnosis of benign strictures was made if all tissue sampling methods produced negative results, no tumor was evident on imaging, and the patient showed no deterioration during the clinical course after a minimum of 6 months of follow‐up.

## CASE 1

**FIGURE 2:**
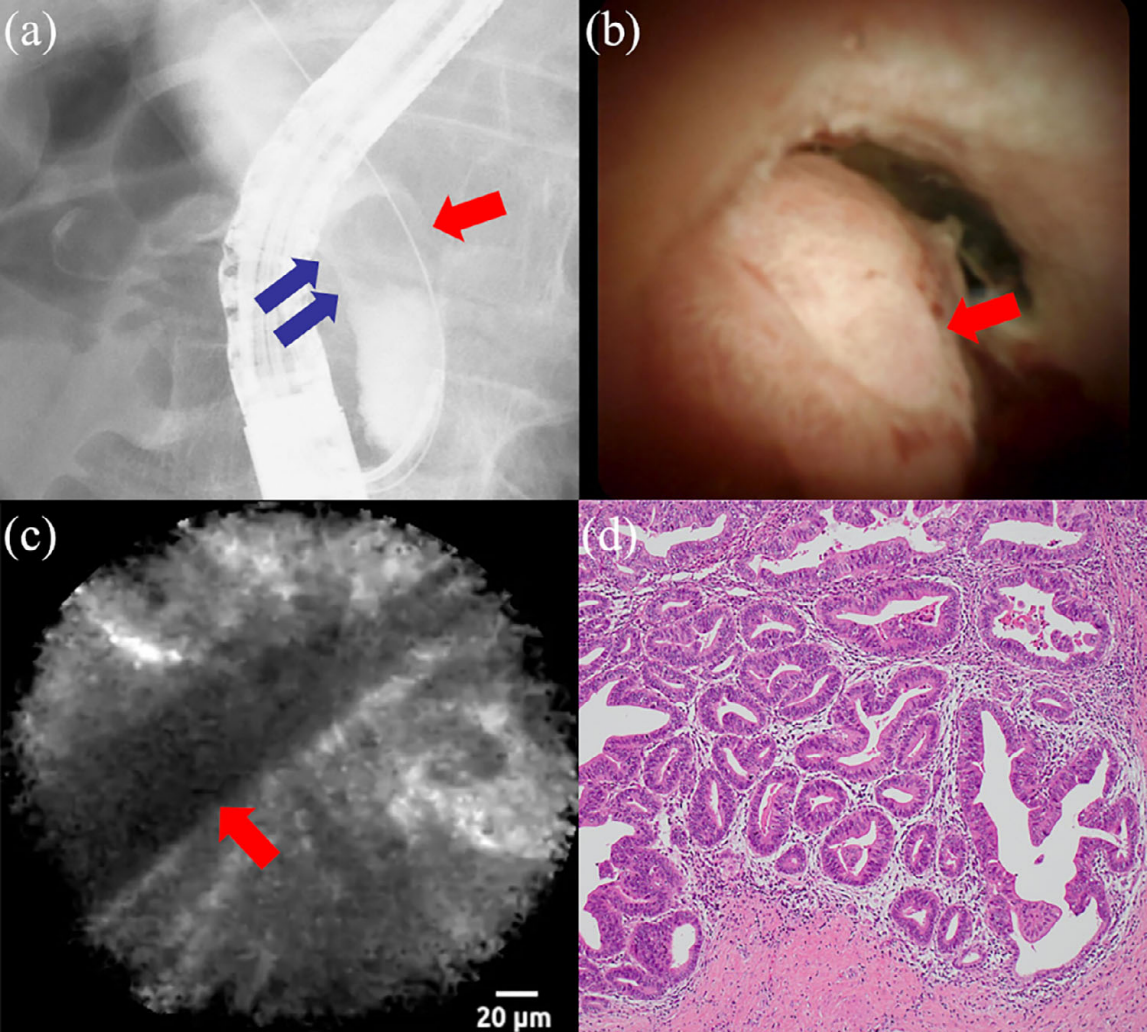
Findings in Case 1. (a) Cholangiography revealing a defect (red arrow) in the distal bile duct and causing biliary stricture (blue arrow). (b) Cholangioscopy revealing a protruding lesion (red arrow) in the distal bile duct. (c) pCLE revealing “thick dark bands” (red arrow) indicating malignancy. (d) Histological findings from a surgical specimen showing adenocarcinoma (H&E, original magnification, ×200). pCLE, probe‐based confocal laser endomicroscopy; H&E, hematoxylin and eosin.

A man in his 80s with jaundice underwent ERCP for detailed inspection. Cholangiography revealed a defect causing a stricture in the distal bile duct (Figure 2a). The histopathological findings from fluoroscopy‐guided biopsy showed no signs of malignancy. As malignancy could not be ruled out, pCLE was performed 2 weeks later (Video S1).

After cholangiography, a cholangioscope was inserted into the bile duct. POCS revealed a protruding lesion in the distal bile duct (Figure 2b). Subsequently, pCLE under direct cholangioscopic visualization was performed, which revealed “thick dark bands” indicating malignancy (Figure 2c). Finally, a POCS‐guided biopsy was performed. The histopathological findings indicated malignancy and a pancreaticoduodenectomy was performed. Histological examination of the surgical specimen revealed adenocarcinoma (Figure 2d).

## CASE 2

A man in his 70s with distal bile duct stricture underwent ERCP at another hospital; however, no definitive diagnosis was made. Therefore, pCLE was performed at our facility (Video S2).

**FIGURE 3:**
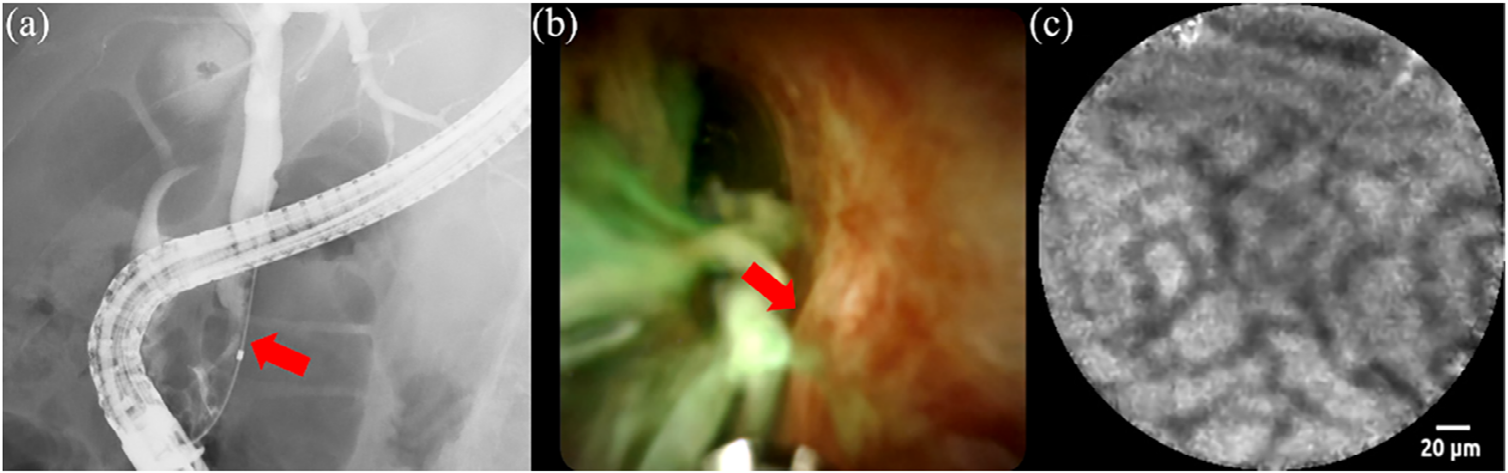
Findings in Case 2. (a) Cholangiography revealing a biliary stricture (red arrow) in the distal bile duct. (b) Cholangioscopy revealing the biliary stricture (red arrow) without any sign of malignancy. (c) pCLE revealing a “reticular network of thin dark branching bands,” indicating a benign stricture. pCLE, probe‐based confocal laser endomicroscopy.

Cholangiography revealed a stricture in the distal bile duct (Figure 3a). POCS also revealed biliary strictures; however, no malignant findings, such as irregular mucosa were observed (Figure 3b). pCLE under direct cholangioscopic visualization revealed a “reticular network of thin dark branching bands” indicating benign lesions (Figure 3c). Histological findings from the POCS‐guided biopsy showed no malignancy. After pCLE, we conducted ERCP‐related procedures, including biopsies from the stricture, twice to confirm if malignancy could be ruled out. The biopsy findings indicated no malignancy. Although various benign biliary strictures, such as primary sclerosing cholangitis and immunoglobulin 4‐related sclerosing cholangitis were considered, we could not detect the specific cause of the stricture. We confirmed no deterioration based on laboratory tests and computed tomography results for the last year. The patient has been followed up without the need for biliary drainage. We believed this biliary stricture was not malignant and could be monitored.

## CASE 3

**FIGURE 4:**
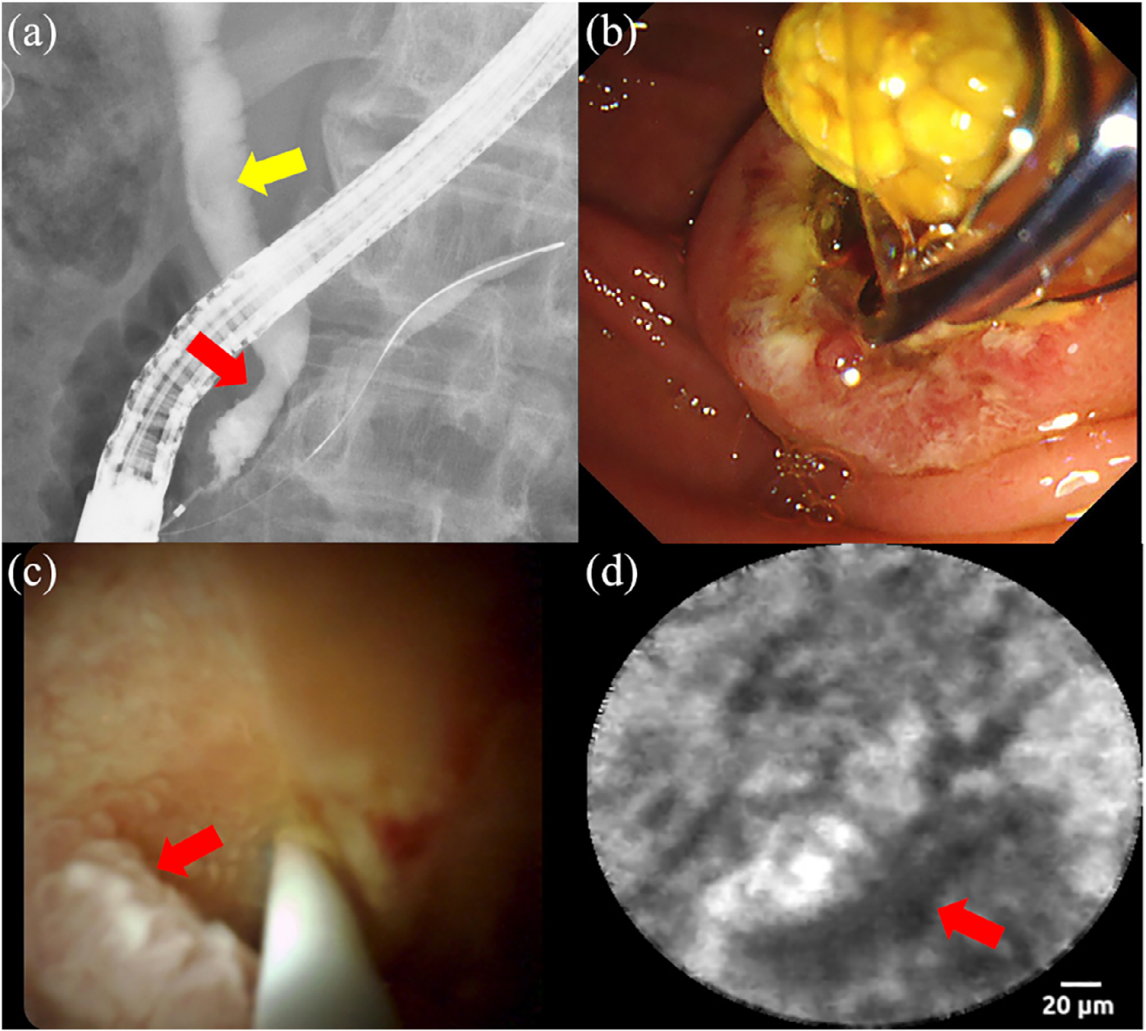
Findings in Case 3. (a) Cholangiography revealing a stone (yellow arrow) and biliary stricture (red arrow) in the distal bile duct. (b) Endoscopic findings revealing successful stone extraction. (c) Cholangioscopy revealing a papillogranular surface without any irregularity (red arrow). (d) pCLE revealing a “thickened reticular structure” (red arrow), indicating inflammation. pCLE, probe‐based confocal laser endomicroscopy.

A man in his 70s with bile duct stones and biliary strictures underwent ERCP. Cholangiography revealed a stone and stricture in the distal bile duct (Figure 4a). Stone extraction and fluoroscopy‐guided biopsy from the stricture site were performed (Figure 4b). Although the histopathological findings showed no signs of malignancy, it could not be ruled out. Therefore, pCLE was performed 2 weeks later (Video S3).

POCS revealed a papillogranular surface without irregularities, indicating benign lesions (Figure 4c). pCLE under direct cholangioscopic visualization revealed a “thickened reticular structure” indicating inflammation (Figure 4d). The histological findings from the POCS‐guided biopsy showed no signs of malignancy. Six months later, we confirmed no deterioration in the clinical course. We diagnosed it as an inflammatory biliary stricture associated with bile duct stones.

## DISCUSSION

Although our case series’ limited number of patients is a substantial limitation, it also has several strengths. All patients underwent pCLE under direct cholangioscopic visualization. This allowed us to apply the pCLE mini‐probe accurately to the areas we wanted to examine. Furthermore, to our knowledge, this is the first case series to focus on the efficacy of pCLE under direct cholangioscopic visualization for biliary strictures that cannot be definitively diagnosed using ERCP. We believe that this case series is the first step in determining if pCLE under direct cholangioscopic visualization is helpful for making an accurate diagnosis in difficult cases.

In this case series, pCLE findings were obtained in three cases, providing accurate diagnosis, and no adverse event was reported. In case 1, the protruding lesion was clearly identified through POCS, and pCLE findings that met the malignancy classification were obtained. In cases 2 and 3, because malignancy could not be ruled out during the initial ERCP, we performed pCLE under direct cholangioscopic visualization, which showed no signs of malignancy. The pCLE findings along with POCS findings helped confirm the diagnosis of benign strictures. Since the specific etiology causing the biliary stricture was not identified in case 2, we will continue to monitor this patient carefully.

Obtaining POCS and pCLE findings simultaneously increases the diagnostic yield in challenging cases. Furthermore, bile duct cancer with progression beneath the surface of the mucosa is difficult to detect with cholangioscopic findings alone, as malignant findings are barely identified on the surface of the mucosa.^10^ In such cases, pCLE diagnosis under cholangioscopy would be a good indication. However, there were several limitations to performing pCLE under direct cholangioscopic visualization. Similar to biopsy under direct cholangioscopic visualization, pCLE under direct cholangioscopic visualization from the distal bile duct, especially just above the papilla, is difficult.^4^ Furthermore, since the cholangioscope is approximately 10Fr, pCLE under direct cholangioscopic visualization is difficult to perform from the peripheral intrahepatic bile duct. In such a case, fluoroscopic‐guided pCLE using a cannulation catheter is favorable.

Since we finished evaluating and diagnosing all three cases in May 2024, we are going to finalize this research.

This case series had several limitations. First, the number of cases was limited; thus, demonstrating the efficacy of pCLE under the direct view of cholangioscopy for the diagnosis of biliary stricture, as noted in our research plan of jRCTs031230238 with only three cases is actually impossible. Second, although the cases might demonstrate the feasibility of pCLE under direct cholangioscopic visualization for biliary strictures that cannot be definitively diagnosed through ERCP using fluoroscopy, biliary strictures that cannot be definitively diagnosed through ERCP using POCS should also be evaluated; this highlights an important area for future research.

In conclusion, pCLE under direct cholangioscopic visualization for indeterminate biliary strictures might be feasible and safe, even though these strictures were not previously diagnosed using ERCP. Further studies are warranted to clarify the effectiveness of pCLE under direct cholangioscopic visualization.

## CONFLICT OF INTEREST STATEMENT

None.

## ETHICS STATEMENT

**‐Approval** of **the research protocol by an Institutional Reviewer Board**.

This research was approved by the Certified Clinical Research Review Committee (number: 232001).

**‐Informed Consent**.

Written informed consent was obtained from each patient before the procedure.

**‐Registry and the Registration No. of the study/trial**.

It has been registered in the Japan Registry of Clinical Trials (jRCT; number: jRCTs031230238).

**‐Animal Studies**.

N/A.

## Supporting information


**TABLE S1** The Miami and Paris classifications for pCLE findings.^8^, ^9^

**VIDEO S1** pCLE under direct cholangioscopic visualization in Case 1.
pCLE, probe‐based confocal laser endomicroscopy.

**VIDEO S2** pCLE under direct cholangioscopic visualization in Case 2.
pCLE, probe‐based confocal laser endomicroscopy.

**VIDEO S3** pCLE under direct cholangioscopic visualization in Case 3.
pCLE, probe‐based confocal laser endomicroscopy.
