# Supplementary material for: Prospective assessment of probe‐based confocal laser endomicroscopy under direct cholangioscopic visualization for biliary strictures that could not be definitively diagnosed using endoscopic retrograde cholangiopancreatography (with video)
Source: DEN Open. 2024 Sep 25;5(1):e70007. doi: 10.1002/deo2.70007 (PMC11424493; doi:10.1002/deo2.70007)
Supplement: Supplementary file 1 — TABLE S1 The Miami and Paris classifications for pCLE findings. 8 , 9 [file DEO2-5-e70007-s002.docx]

Supplementary Table S1. The Miami and Paris classifications for pCLE findings.^8,9^

| Malignancy |
| --- |
| 1. Thick white bands (>20 µm) |
| 2. Thick dark bands (>40 µm) |
| 3. Dark clumps |
| 4. Epithelium |
| Inflammation |
| 1. Thickend reticular structure |
| 2. Increased inter-glandular space |
| 3. Roughness aspect |
| 4. Vascular congestion |
| Benign |
| 1. Reticular network of thin dark branching bands (<20 µm) |
| 2. Light-gray background |
| 3. Blood vessels (<20 µm) |

pCLE, probe-based confocal laser endomicroscopy
